# Variations in Acetabular Offset Between the Preoperative Plan (mediCAD^®^) and Postoperative Radiographic Measurements: an overlooked source of error that affects restoration of global offset after THA

**DOI:** 10.1051/sicotj/2026050

**Published:** 2026-08-07

**Authors:** Anatole Matray, Clément Cazemajou, Tanguy Vendeuvre, Pierre Pries, Guillaume Anthony Odri, Mathieu Severyns

**Affiliations:** 1 Department of Orthopaedic Surgery, CHU de Poitiers 86000 Poitiers France; 2 Department of Orthopaedic Surgery, CH La Rochelle 17000 La Rochelle France; 3 Department of Orthopaedic Surgery, CHU d'Orléans 45100 Orléans France; 4 Department of Orthopaedic Surgery, Clinique Porte Océane, Centre Sablais de l'appareil Locomoteur (CESAL) 85340 Les Sables d'Olonne France

**Keywords:** Total hip arthroplasty, Radiographic measurement, Acetabular offset

## Abstract

*Introduction*: Restoring the offset after THA is positively correlated with good functional outcomes and patient satisfaction. Preoperative planning is now an essential tool for optimizing this restoration. Nevertheless, recent studies have focused mainly on the femoral offset, at the expense of the acetabular offset. Our hypothesis was that the variations in global offset after THA are mainly due to variations in acetabular offset relative to its initially planned value. *Methods*: This retrospective study involved 104 patients who had undergone primary anterior THA with two-dimensional preoperative planning between January 2023 and November 2024. The planned preoperative radiographs and the postoperative radiographs were analyzed independently by two attending orthopeadic surgeons who measured the global, acetabular, and femoral offsets. The intra-rater and inter-rater agreement was determined. The differences between the planned implant sizes and the size actually implanted were also determined. *Results*: In the end, 43 patients met all the pre and postoperative radiographic criteria and were included in the analysis. There was a correlation between the variation in acetabular offset and the variations in global offset (*r* = 0.74, *p* < 0.0001) and between the variation in femoral offset and the variations in global offset (*r* = 0.68, *p* < 0.0001). Using multiple linear regression, the coefficients of regression for the variation in the acetabular offset and femoral offset were 1.00, indicating an equal contribution (50%) to variations in global offset. *Conclusion*: Variations in the positioning of the acetabular cup are an important factor in the variations in global offset after THA. The postoperative variations in acetabular and femoral offsets contribute equally to changes in the global offset. Clinically, this means that practitioners should focus more on implanting the acetabular component. *Registration*: IRB approval N° 22727513. *Level of evidence*: III.

## Introduction

The aim of total hip arthroplasty (THA) is to relieve the patient’s pain while restoring the anatomy of the hip joint, namely its femoral center of rotation [1–3]. In fact, restoring the offset after THA is positively correlated with good functional outcomes and patient satisfaction.

Preoperative planning is now frequently done to determine the size and position of the implants with the aim of restoring the center of rotation during the surgical procedure [4]. It helps with planning the height of the femoral cut, the depth of acetabular reaming, and the size of the various implanted components. Two-dimensional (2D) preoperative planning using mediCAD^®^ [5] software appears to provide good reproducibility in the positioning and choice of implants, allowing the femoral offset to be restored after THA [6]. Nevertheless, the majority of published studies focus on the femoral offset, while few studies have looked into restoring the acetabular offset [7]. We believe the acetabular offset may be more variable due to the anatomical variations observed in the acetabulum and the lack of visual landmarks during reaming of the acetabulum and positioning of the chosen acetabular cup [8].

We hypothesized that the variations in global offset after THA are mainly due to variations in acetabular offset relative to its initially planned value. The primary aim of this study was to compare the pre-planned position of the acetabular cup with its final position postoperatively. The secondary aim was to evaluate the impact of variations in acetabular offset on the global offset between the 2D preoperative plan and postoperative radiographic measurements.

## Methods

### Study design

This was a retrospective analysis of data collected prospectively from patients who underwent THA by a single surgeon. The inclusion criteria were patients who underwent primary THA with preoperative planning done using the mediCAD^®^ software, no matter their age, who had postoperative control radiographs, and who provided their consent for analysis of radiographic data. The exclusion criteria were poor quality pre- or postoperative radiographs, radiographic views that do not encompass the contralateral hip, pubic symphysis, and sacrum; no calibration marker on the preoperative radiographs; THA done for femoral neck fracture; and the patient declined to participate. The study was approved by our institutional review board (IRB 22727513).

### Study population

**Figure 1:**
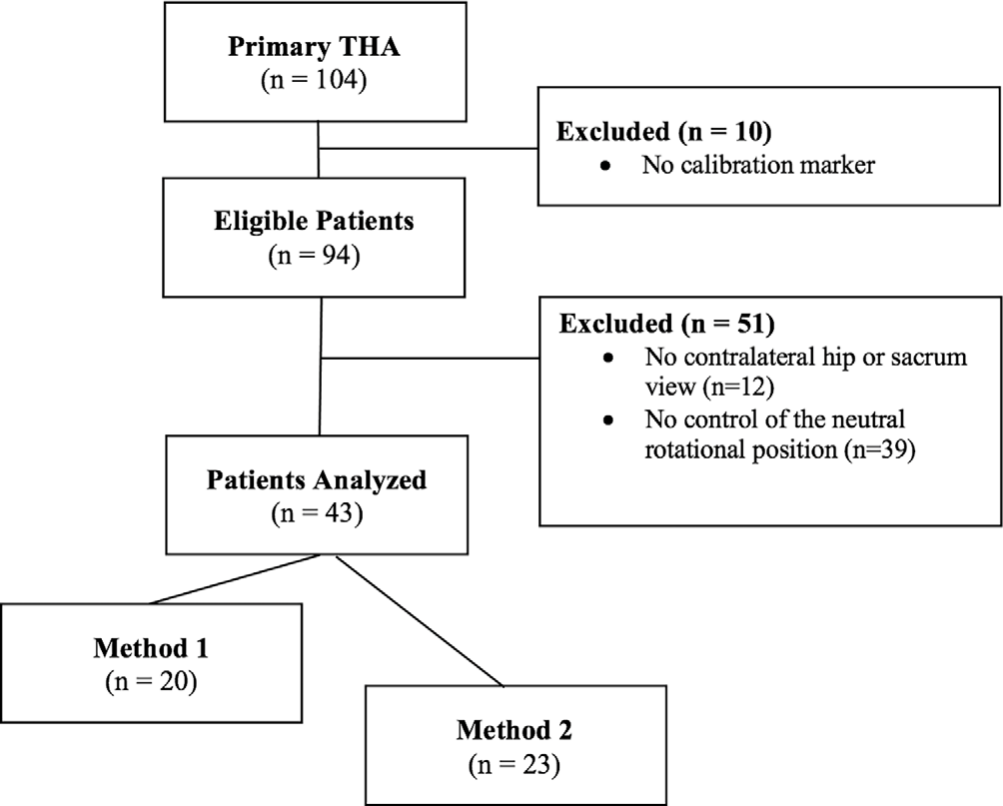
Flowchart of the 104-patient cohort.

In all, 104 patients had undergone THA between January 2023 and November 2024 and had at least 3 months of follow-up. Ten patients were excluded because there was no calibration marker on their radiographs, while 51 were excluded because the radiographs were not of sufficiently high quality (Figure 1).

The surgical indication was determined during the preoperative consultation, which consisted of medical history, a full clinical exam of the hip, and a radiographic work-up with AP and lateral views of the hip. Additional imaging with a calibration marker was done on an AP view of the hip or pelvis to carry out preoperative planning with the mediCAD^®^ 2D software (mediCAD^®^, Altdorf, Germany) of the THA components to be implanted.

### Surgical technique

**Figure 2:**
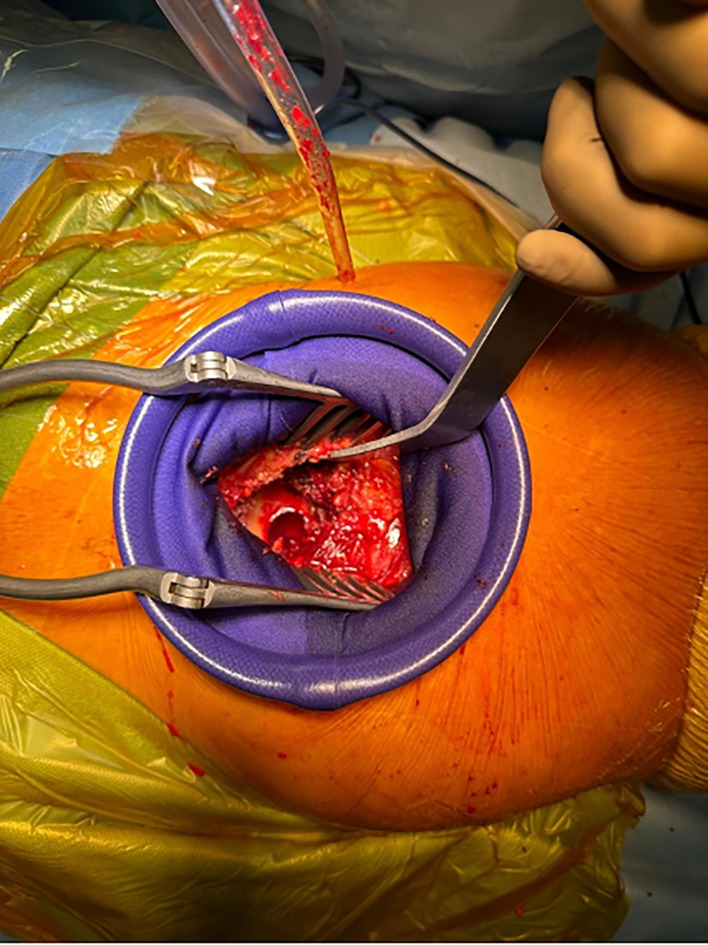
Acetabular exposure with Alexis^®^ wound protector-retractor.

A Hueter-Gaine approach was used with the patient supine on a standard operating table. After making an 8-cm long incision, the aponeurosis of the tensor fascia lata was opened, and its muscle belly was reflected posteriorly and laterally. The ascending branches of the lateral circumflex femoral artery were identified and carefully ligated, exposing the joint capsule. The femoral neck and acetabulum were exposed with a Merle d’Aubigné retractor and Beckmann retractor. For the femoral phase, the leg was placed in adduction and external rotation, and a femoral neck elevator was applied under the greater trochanter to make it easier to insert the broaches. Throughout the procedure, the skin was protected with a commercial device (Alexis^®^ Wound Protector-Retractor HR000, Applied Medical, USA) (Figure 2).

All patients received a UTS^TM^ short cementless femoral stem (United, Taiwan) and a U-MOTION II+^TM^ press-fit acetabular cup (United). A ceramic-ceramic bearing was used. Its exact size was determined during the intraoperative trials, with the surgeon selecting the appropriate acetabular cup size and choosing between the available head diameters (28, 32, or 36 mm) and neck lengths (short, medium, long, extra-long). The industry partner, which also provides the two-dimensional planning software, exclusively offers prostheses with either standard or lateralized offset.

### Data collection

The preoperative planning radiographs and postoperative radiographs were analyzed independently by two attending surgeons. Radiographs that met quality standards [9] were retained for the analysis. The AP views of the pelvis were classified into group 1, and the AP views of the hip that captured the pubic symphysis and sacrum were classified into group 2. The planned and postoperative offsets were measured for each patient. The acetabular offset was the distance between the femoral head center of rotation and a perpendicular line across the acetabular teardrops (trans-teardrop line). The femoral offset was the distance between the intramedullary axis of the femoral shaft and the center of rotation of the femoral head. The global offset was defined as the sum of the femoral offset, which represents the distance between the femoral axis and the femoral center of rotation, and the acetabular offset, defined as the distance between the femoral center of rotation and the acetabulum.

**Figure 3:**
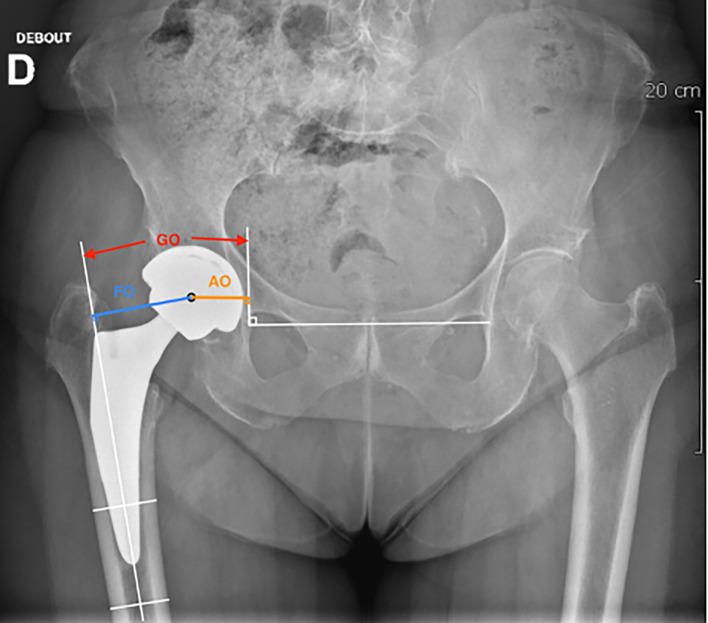
Method 1: postoperative offset measurement after total hip arthroplasty (AO: Acetabular Offset; FO: Femoral Offset; GO: Global Offset).

**Figure 4:**
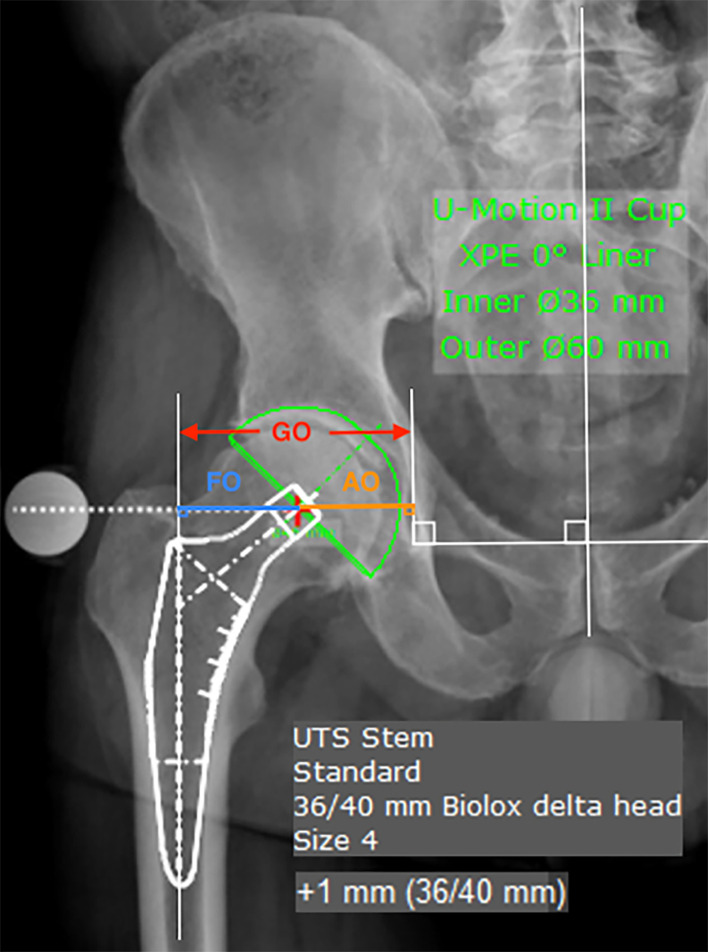
Method 2: 2D templating to measure offsets (AO: Acetabular Offset; GO: Global Offset; FO: Femoral Offset).

For the radiographs in group 1, the teardrop line was drawn directly to allow calculation of the acetabular offset [10] (Method 1) (Figure 3), while for the radiographs in group 2, it was drawn perpendicular to the line passing through the axis of the sacrum spinous processes and the center of the pubic symphysis [8, 11] (Method 2) (Figure 4).

### Data analysis

The statistical analysis was performed using JMP software (version 7.0.1, SAS Institute). The distribution of continuous variables was evaluated with a Shapiro-Wilk test. Normally distributed data were summarized by the mean and SD values and compared using parametric tests. Comparisons between pre- and postoperative data were done using a paired Student *t* test. Qualitative variables were expressed as counts and percentages.

The consistency of the radiographic measurements (acetabular offset, femoral offset, and global offset) was evaluated by calculating the intraclass correlation coefficient (ICC) using a two-factor random effects model, with absolute agreement:

- ICC(2,1) for intra-rater reliability (measurements 1 and 2 for each surgeon)
- ICC(2,k) for the inter-rater reliability (mean of measurements by the two surgeons).

The ICC values were interpreted using the standard thresholds: <0.5 low, 0.5–0.75 moderate, 0.75–0.9 good, >0.9 excellent.

The Pearson test was used to determine the strength of a correlation between two variables. The relationship between variations in offset (Δ = postoperative value – planned value) was explored using bivariate analyses, then multiple linear regressions when several explanatory variables were included simultaneously. A partial ANOVA and Shapley values were used to estimate how much each variable contributed to the variance.

The significance threshold was set at *p* < 0.05.

## Results

### Study population

The characteristics of the study population are summarized in Table 1.

**Table 1:** Patient characteristics.

Variable	Value
Age at surgery, mean ± SD (min–max) (*y*)	75.1 ± 11.7 (47–94)
Sex, *n* (%)	
Men	19 (44%)
Women	24 (56 %)
Hip right/left No. (%)	28 (65%) / 15 (35%)
Method	
1	20
2	23

*n*: number; SD: standard deviation.

### Correlation of offsets

There was a significant correlation between the postoperative global offset and planned global offset (*r* = 0.62, *p* < 0.0001), the postoperative femoral offset and planned femoral offset (*r* = 0.60, *p* < 0.0001), and the postoperative acetabular offset and planned acetabular offset (*r* = 0.35, *p* = 0.023). The bivariate analysis found a correlation between the variation in acetabular offset and variations in global offset (*r* = 0.74, *p* < 0.0001).

There was also a correlation between the variation in femoral offset and variations in global offset (*r* = 0.68, *p* < 0.0001).

Based on the multiple linear regression, the regression coefficients for the variation in the acetabular offset and femoral offset were 1.00, showing equal contributions (50% each) to the variations in global offset. This analysis found no correlation between the variation in acetabular offset and the variation in femoral offset.

### Consistency between planned and implanted THA components

The accuracy of the preoperative planning is shown in Table 2.

**Table 2:** Agreement between the planned size and implanted size.

	Exact cup size	Cup ±1 size	Exact stem size	Stem ±1 size	Head size	Neck size
Agreement (%)	49	74	44	82	86	47

### Validity of measurement methods

The ICC values, evidence of intra- and inter-rater reliability, were rated as good or excellent (Table 3).

**Table 3:** Intraclass Correlation Coefficient for intra-rater and inter-rater reliability.

	Acetabular offset		Femoral offset		Global offset	
	Planned	Post-op	Planned	Post-op	Planned	Post-op
Intra-rater						
Surgeon 1	0.90	0.84	0.75	0.85	0.91	0.84
Surgeon 2	0.78	0.86	0.94	0.89	0.89	0.84
Inter-rater	0.94	0.90	0.99	0.94	0.98	0.88

Given the good intra- and inter-rater reliability, the mean of the four values (2 observations by 2 raters) was used. The values are the mean for all the patients (in mm) with SD. There was no significant difference between the mean planned offset and the mean postoperative offset.

## Discussion

This study shows that the acetabular cup’s positioning has the same impact on postoperative offset as the femoral stem’s positioning. It highlights the importance of acetabular positioning and preoperative planning, which accurately predict the implant size and positioning, partially confirming our hypothesis.

We were interested in variations in the acetabular offset, as there are fewer studies on this element than on the femoral offset [11]. The acetabular cup’s positioning depends on the depth and height of reaming, which must be adjusted for each patient, combining optimal coverage, orientation, and hold of the implant [12]. It varies based on the patient’s anatomy, surgical approach, and components used [13, 14]. A hip with normal anatomy has more positioning possibilities. In cases of defective anterior or lateral coverage, the acetabular implant must be medialized; the femoral offset must compensate for this to maintain a satisfactory global offset [15]. Despite the lesser emphasis on acetabular offset in scientific literature compared to femoral offset, restoring it appears to be an important factor affecting the restoration of various offsets during THA, having clinical repercussions on the patient’s gait kinetics [15].

We did this study to understand the effect of acetabular offset on the global offset. We found that variations in global offset are explained as much by the variations in femoral offset as by the variations in acetabular offset. There was no correlation between the variation in acetabular offset and the variation in femoral offset, which means they vary independently. We believe this is a novel finding. These results can be explained by the fact that the surgeon did intraoperative trials, adjusting the length of the femoral neck to restore an optimal clinical global offset, which makes it possible to compensate for differences between the planned and final components. The variations between the neck size planned and the one implanted were actually larger than for the other components.

Despite recent published data that 3D images provide better accuracy for the reproducibility of chosen component sizes [16, 17]. We chose to use a 2D planning method because the images obtained by CT scan are not superior when measuring acetabular offset [6]. The other advantages of 2D planning are that there is less radiation, the cost is lower, and it is easier to carry out in current practice.

The accuracy of the planning for component sizes and restoring the various offsets is similar to other studies using mediCAD^®^ software, which shows the dependability of this software package during the planning process [4]. Given this good pre- and postoperative reliability, we believe that preoperative planning is crucial as it helps to reduce surgery time [18], improves surgical precision, and reduces procedure-related costs [19]. The radiographic measurement methods used here were derived from published data and allowed us to adapt the analysis to the variable quality of the postoperative radiographs [20–22]. Since the psychometric qualities of the measurement methods have not been validated, we calculated the intra-rater and inter-rater consistency. The ICC values were good or excellent, which allowed us to be confident in the results and highlight their usefulness in current practice.

It is important to note that recent studies have evaluated the restoration of global offset using navigation or robotic assistance [23, 24]. These studies have mainly been conducted with anterior surgical approaches [25]: the use of robotics for THA by DAA provided an advantage in controlling the orientation of the cup and the restoration of its rotation center. Thanks to the 3D planning on the CT scan, it allowed us to respect the thresholds while avoiding the anterior overhangs. These findings are important to consider from the perspective of further democratizing the use of robotic assistance.

All the patients were operated on by a single surgeon, which ensures the planning technique is fully mastered and the implantation technique is highly reproducible. This study has certain limitations. Many patients were excluded because their radiographs did not meet quality standards. Consequently, two measurement methods were used to adapt to the variable quality of the radiographs. Our study focused solely on radiographic analysis of variations in offsets in patients undergoing THA; it did not determine the clinical impact of these variations, especially in the occurrence of postoperative complications (dislocation, loosening, etc.). Thus, it would be important to investigate whether restoring the global offset after THA using our planning tool produces satisfactory long-term functional outcomes.

## Conclusion

The variations in positioning of the acetabular cup are an important factor in variations of the global offset after THA. The postoperative variations in acetabular and femoral offset equally contribute to the variations in global offset. This should motivate clinicians to pay close attention to the implantation of the acetabular cup. To supplement this immediate postoperative radiographic analysis, the clinical impact of these variations in global offset due to variations in acetabular offset should be determined in patients undergoing THA.

## Data Availability

This article has no associated generated data.

## References

[1] Ferguson RJ, Palmer AJ, Taylor A, Porter ML, Malchau H, Glyn-Jones S (2018) Hip replacement. Lancet 392, 1662–1671. 30496081 10.1016/S0140-6736(18)31777-X

[2] Bendaya S, Anglin C, Lazennec J-Y, Allena R, Thoumie P, Skalli W (2016) Good vs poor results after total hip arthroplasty: An analysis method using implant and anatomic parameters with the EOS imaging system. J Arthroplasty 31, 2043–52. 27297114 10.1016/j.arth.2015.12.036

[3] Snell DL, Dunn JA, Hooper G (2024) Associations between pain, function and quality of life after total hip arthroplasty. Int J Orthop Trauma Nurs 54, 101121. 39029151 10.1016/j.ijotn.2024.101121

[4] Montiel V, Troncoso S, Valentí-Azcárate A, Valentí-Nin JR, Lamo-Espinosa JM (2020) Total hip arthroplasty digital templating: Size predicting ability and interobserver variability. Indian J Orthop 54, 840–847. 33133407 10.1007/s43465-020-00217-0 PMC7572938

[5] Mirghaderi SP, Sharifpour S, Moharrami A, et al. (2022) Determining the accuracy of preoperative total hip replacement 2D templating using the mediCAD^®^ software. J Orthop Surg Res 17, 222. 35399090 10.1186/s13018-022-03086-5 PMC8996579

[6] Tone S, Hasegawa M, Naito Y, Wakabayashi H, Sudo A (2022) Comparison between two- and three-dimensional methods for offset measurements after total hip arthroplasty. Sci Rep 12, 12644. 35879390 10.1038/s41598-022-16952-3 PMC9314396

[7] Lecerf G, Fessy MH, Philippot R, et al. (2009) Femoral offset: Anatomical concept, definition, assessment, implications for preoperative templating and hip arthroplasty. Orthop Traumatol Surg Res 95, 210–219. 19423418 10.1016/j.otsr.2009.03.010

[8] Luca DiGiovanni P, Gasparutto X, Armand S, Hannouche D (2023) The modern state of femoral, acetabular, and global offsets in total hip arthroplasty: a narrative review. EFORT Open Rev 8, 117–126. 36916758 10.1530/EOR-22-0039 PMC10026057

[9] Godefroy D, Chevrot A, Morvan G, Rousselin B, Sarazin L (2008) Plain films of pelvis. J Radiol 89, 679–690. 18535514 10.1016/s0221-0363(08)71503-8

[10] Dastane M, Dorr LD, Tarwala R, Wan Z (2011) Hip offset in total hip arthroplasty: quantitative measurement with navigation. Clin Orthop Relat Res 469, 429–436. 20844997 10.1007/s11999-010-1554-7 PMC3018189

[11] Bonnin MP, Archbold PHA, Basiglini L, Fessy MH, Beverland DE (2012) Do we medialise the hip centre of rotation in total hip arthroplasty? Influence of acetabular offset and surgical technique. Hip Int 22, 371–378. 22865253 10.5301/HIP.2012.9350

[12] Bhaskar D, Rajpura A, Board T (2017) Current Concepts in Acetabular Positioning in Total Hip Arthroplasty. Indian J Orthop 51, 386–96. 28790467 10.4103/ortho.IJOrtho_144_17 PMC5525519

[13] Weber M, Merle C, Nawabi DH, Dendorfer S, Grifka J, Renkawitz T (2020) Inaccurate offset restoration in total hip arthroplasty results in reduced range of motion. Sci Rep 10, 13208. 32764592 10.1038/s41598-020-70059-1 PMC7413373

[14] Murray DW (1993) The definition and measurement of acetabular orientation. J Bone Joint Surg Br 75, 228–232. 8444942 10.1302/0301-620X.75B2.8444942

[15] Esbjörnsson A-C, Kiernan S, Mattsson L, Flivik G (2021) Geometrical restoration during total hip arthroplasty is related to change in gait pattern – a study based on computed tomography and three-dimensional gait analysis. BMC Musculoskelet Disord 22, 369. 33879123 10.1186/s12891-021-04226-4 PMC8058981

[16] Parisi FR, Zampogna B, Zampoli A, et al. (2024) Planning accuracy and stem offset assessment in digital two-dimensional versus three-dimensional planning in cementless hip arthroplasty: A systematic review and meta-analysis. JCM 13, 6566. 39518705 10.3390/jcm13216566 PMC11546058

[17] Aubert T, Galanzino G, Gerard P, Le Strat V, Rigoulot G, Lhotellier L (2023) Accuracy of preoperative 3D vs 2D digital templating for cementless total hip arthroplasty using a direct anterior approach. Arthroplast Today 24, 101260. 38023640 10.1016/j.artd.2023.101260 PMC10652126

[18] Vigdorchik JM, Sharma AK, Jerabek SA, Mayman DJ, Sculco PK (2021) Templating for total hip arthroplasty in the modern age. J Am Acad Orthop Surg 29, e208–e216. 33543909 10.5435/JAAOS-D-20-00693

[19] Holzer LA, Scholler G, Wagner S, Friesenbichler J, Maurer-Ertl W, Leithner A (2019) The accuracy of digital templating in uncemented total hip arthroplasty. Arch Orthop Trauma Surg 139, 263–268. 30523444 10.1007/s00402-018-3080-0 PMC6373540

[20] Doehrmann R, Comer BJ, Chatterji R, Diedring B, Knapp P, Afsari A (2023) Accuracy of leg length and hip offset measurements using a fluoroscopic grid during anterior approach total hip arthroplasty. Arthroplast Today 22, 101154. 37502102 10.1016/j.artd.2023.101154 PMC10369392

[21] Austin DC, Dempsey BE, Kunkel ST, Torchia MT, Jevsevar DS (2019) A comparison of radiographic leg-length and offset discrepancies between 2 intraoperative measurement techniques in anterior total hip arthroplasty. Arthroplast Today 5, 181–186. 31286041 10.1016/j.artd.2018.09.005 PMC6588659

[22] Fey B, Brenneis M, Stief F, van Drongelen S (2024) Effect of stem design and positioning on the leg axis after total hip arthroplasty: A secondary analysis. J Clin Med 13, 4453. 39124720 10.3390/jcm13154453 PMC11313081

[23] Van Ravestyn A, Frantz T, Vandemeulebroucke J, Jansen B, Duerinck J, Scheerlinck T (2024) Determination of rotation center and diameter of femoral heads using off-the-shelf augmented reality hardware for navigation. Sci Rep 14(1), 15458. 38965266 10.1038/s41598-024-64957-x PMC11224340

[24] Shimizu A, Murakami S, Tamai T, Haga Y, Kutsuna T, Kinoshita T, Takao M (2025) Comparing cup placement, leg length, and offset discrepancy after total hip arthroplasty between CT-based robotic arm-assisted and navigation systems. Bone Jt Open 6(1), 3–11. 39740687 10.1302/2633-1462.61.BJO-2024-0173.R1 PMC11688127

[25] Foissey C, Batailler C, Coulomb R, Giebaly DE, Coulin B, Lustig S, Kouyoumdjian P (2023) Image-based robotic-assisted total hip arthroplasty through direct anterior approach allows a better orientation of the acetabular cup and a better restitution of the center of rotation than a conventional procedure. Int Orthop 47(3), 691–699. 36348089 10.1007/s00264-022-05624-6

